# International Expansion of a Novel SARS-CoV-2 Mutant

**DOI:** 10.1128/JVI.00567-20

**Published:** 2020-06-01

**Authors:** Minjin Wang, Mengjiao Li, Ruotong Ren, Lifeng Li, En-Qiang Chen, Weimin Li, Binwu Ying

**Affiliations:** a Department of Laboratory Medicine, West China Hospital of Sichuan University, Chengdu, China; b Department of Hematology, The First Hospital of Lanzhou University, Lanzhou, China; c Genskey Biotechnology Co., Ltd., Beijing, China; d Center of Infectious Diseases, West China Hospital of Sichuan University, Chengdu, China; e Department of Respiratory and Critical Care Medicine, West China Hospital of Sichuan University, Chengdu, China; University of California, Irvine

**Keywords:** SARS-CoV-2, molecular epidemiology, mutation, virus genome

## LETTER

RNA viruses such as coronavirus are rapidly evolving pathogens that can accumulate considerable genetic diversity in relatively short time periods. Mutation accumulated in severe acute respiratory syndrome coronavirus 2 (SARS-CoV-2) genomes during its pandemic spread can cause unpredictable effects on coronavirus disease 2019 (COVID-19) and further complicate epidemic control efforts (1). Here we report that a novel SARS-CoV-2 mutation in its ORF3a gene appears to be spreading worldwide, which deserves close attention.

We collected 95 SARS-CoV-2 samples from the Sichuan Province of China for amplification-free whole-genome sequencing and acquired 13 whole-genome sequences, which were analyzed for sequence variation and evolution together with 199 SARS-CoV-2 genomes publicly released in the GISAID EpiFlu database (https://www.gisaid.org/) (2) and 7 genomes downloaded from the National Genomics Data Center (NGDC) database (https://bigd.big.ac.cn/ncov). This study was approved by the Biomedical Research Ethics Committee of the West China Hospital of Sichuan University (reference no. 193, 2020) with a waiver of informed consent.

**FIG 1:**
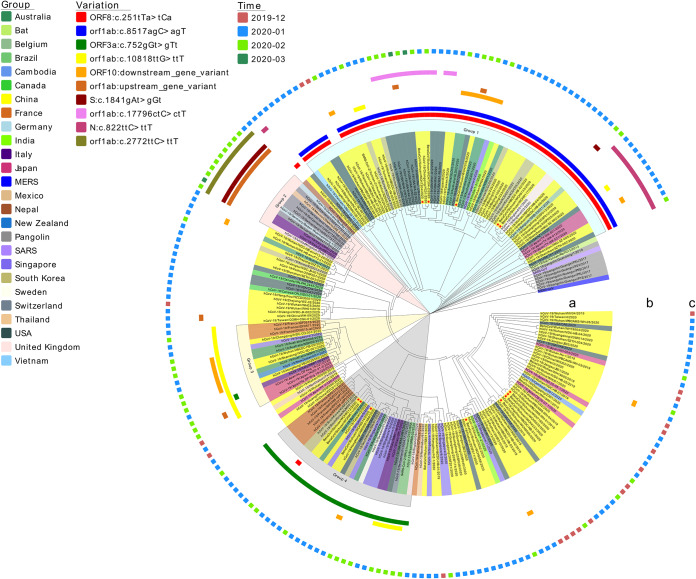
Maximum likelihood tree based on the whole-genome sequences of 221 viral strains. A total of 199 high-quality genomes were collected from the GISAID EpiFlu database, including 1 Rhinolophus affinis isolate, 6 Manis javanica isolates, and 2 environmental isolates. Twenty-two additional genomes were collected from other resources, including 7 genomes from NGDC (https://bigd.big.ac.cn/ncov) and 13 genomes from the West China Hospital (WCH) of Sichuan University. SARS-CoV (NC_004718.3) and Middle East respiratory syndrome coronavirus (MERS-CoV) (NC_019843.3) genome sequences were downloaded from the NCBI RefSeq database. MAFFT (version 7.543) was used for sequence alignment, and PhyML (version 3.0) was used to construct the evolutionary tree. Variation information on the human SARS-CoV-2 genome was derived from NGDC. Mutations of 13 WCH genomes were analyzed using NGDC online tools (https://bigd.big.ac.cn/ncov/tool/variation-identify). Variations were displayed in a unified format: gene: c. position of the variant nucleotide in the coding sequence and the sequence change information, e.g., ORF8: c. 251tTa>tCa. Uppercase letters represent the variant nucleotides.

Based on 10 high-frequency mutations (mutant allele frequency of >5%), these SARS-CoV-2 genomes can be classified into five main groups: original strain 1 and four variants with different mutations groups and clusters (Fig. 1). The most common variants (group 1) exhibited both a missense mutation (ORF8:c.251tTa>tCa; present in 31.58% of the isolates) and a synonymous mutation (orf1ab:c.8517agC>agT; found in 30.62% of the isolates), suggesting a possible linkage between these two sites. Also, these subgroups evolved in the main group 1 with three other mutations. Group 2 was clustered together with mutants including missense variant S: c.1841gAt>gGt, orf1ab upstream gene variant and synonymous variant orf1ab: c.2772ttC>ttT. Group 3 viral isolates were much less frequent (11.48%) and characterized by a missense mutation (orf1ab:c.10818ttG>ttT). Group 4 viral isolates contained a novel missense mutation (ORF3a:c.752gGt>gTt) first identified in a Chinese family. Notably, however, group 4 viral isolates were most frequently found outside mainland China (23.28%; 27/116; *P* < 0.01 by Fisher’s exact test). Additionally, group 2 and group 4 showed obvious aggregation in non-Chinese countries and regions.

The family (an elderly woman and two young family members) carrying the group 4 variant returned from Wuhan to their hometown in Sichuan on 20 January 2020. By 23 January 2020, the elderly woman exhibited symptoms of fever and cough, and her two children also developed these symptoms in the following days. Their throat swab samples were tested and gave SARS-CoV-2-positive results by reverse real-time PCR assay on 25 January 2020. The elderly woman with chronic hypertension was in critical condition with COVID-19 disease, while the two young family members showed mild symptoms. The underlying disease may have contributed to the progress of the disease. None of these individuals traveled outside of China between the start of the COVID-19 epidemic and their return to Sichuan; however, the group 4 variant has demonstrated global dissemination.

We performed a timeline analysis using the sample collection dates reported in the GISAID EpiFlu database. Except for the three patients from Sichuan, China, who traveled from Wuhan prior to the onset of symptoms, group 4 isolates with ORF3a mutant were subsequently reported in several other countries and regions, including China (Taiwan), France (Paris), Australia (Sydney and Clayton), Singapore, South Korea, United Kingdom, and Italy. It should be noted that this mutant virus strain appears to be the most prevalent form of SARS-COV-2 in France, Italy, Brazil, and Singapore.

Virus genome data from France indicate that SARS-CoV-2 strains carrying ORF3a:c.752gGt>gTt often have a S:c.1099Gtc>Ttc mutation in their S gene, which interacts with ACE2 mediating viral entry into its host cells (3), and is regarded as a critical factor for viral transmission and virulence (4, 5). It is not yet clear whether this mutation is common in group 4 viral isolates from different geographical regions. Given the prevalence of group 4 isolates in multiple countries, including France, Italy, and South Korea, which is experiencing a rapidly growing epidemic, this information should be of significant importance to further investigate whether this mutation enhances host cell entry.

At present, the SARS-CoV-2 epidemic in China is diminishing owing to collected control efforts, but the rapid global spread has become a major health concern. Very little is known about how rapidly the SARS-CoV-2 genome mutates and how this affects transmission or pathogenesis. Our findings indicate that comprehensive studies combining genomic epidemiological, and clinical data urgently need to be performed to clarify these issues.

## References

[1] World Health Organization. 2020 Coronavirus disease (COVID-19) situation reports. World Health Organization, Geneva, Switzerland www.who.int/emergencies/diseases/novel-coronavirus-2019/situation-reports/.

[2] Shu Y, McCauley J 2017 GISAID: global initiative on sharing all influenza data − from vision to reality. Euro Surveill 22(13):pii=30494 http://www.eurosurveillance.org/ViewArticle.aspx?ArticleId=19417. 10.2807/1560-7917.ES.2017.22.13.30494 PMC5388101 28382917

[3] Letko M, Marzi A, Munster V 2020 Functional assessment of cell entry and receptor usage for SARS-CoV-2 and other lineage B betacoronaviruses. Nat Microbiol 5:562–569. doi:10.1038/s41564-020-0688-y. 32094589 PMC7095430

[4] Lu G, Wang Q, Gao GF 2015 Bat-to-human: spike features determining ‘host jump’ of coronaviruses SARS-CoV, MERS-CoV, and beyond. Trends Microbiol 23:468–478. doi:10.1016/j.tim.2015.06.003. 26206723 PMC7125587

[5] Hamming I, Timens W, Bulthuis ML, Lely AT, Navis G, van Goor H 2004 Tissue distribution of ACE2 protein, the functional receptor for SARS coronavirus. A first step in understanding SARS pathogenesis. J Pathol 203:631–637. doi:10.1002/path.1570. 15141377 PMC7167720

